# DeepSplice: a deep learning approach for accurate prediction of alternative splicing events in the human genome

**DOI:** 10.3389/fgene.2024.1349546

**Published:** 2024-06-21

**Authors:** Mohammad Abrar, Didar Hussain, Izaz Ahmad Khan, Fasee Ullah, Mohd Anul Haq, Mohammed A. Aleisa, Abdullah Alenizi, Shashi Bhushan, Sheshikala Martha

**Affiliations:** ^1^ Faculty of Computer Studies, Arab Open University, Muscat, Oman; ^2^ Department of Computer Science, Bacha Khan University Charsadda, Charsadda, Pakistan; ^3^ Computer and Information Sciences department, Universiti Teknologi PETRONAS, Seri Iskandar, Malaysia; ^4^ Department of Computer Science, College of Computer and Information Sciences, Majmaah University, Al-Majmaah, Saudi Arabia; ^5^ Department of Information Technology, College of Computer and Information Sciences, Majmaah University, Al-Majmaah, Saudi Arabia; ^6^ School of Computer Science and Artificial Intelligence, SR University, Warangal, India

**Keywords:** alternative splicing, machine learning, deep learning, CNN, neural networks

## Abstract

Alternative splicing (AS) is a crucial process in genetic information processing that generates multiple mRNA molecules from a single gene, producing diverse proteins. Accurate prediction of AS events is essential for understanding various physiological aspects, including disease progression and prognosis. Machine learning (ML) techniques have been widely employed in bioinformatics to address this challenge. However, existing models have limitations in capturing AS events in the presence of mutations and achieving high prediction performance. To overcome these limitations, this research presents deep splicing code (DSC), a deep learning (DL)-based model for AS prediction. The proposed model aims to improve predictive ability by investigating state-of-the-art techniques in AS and developing a DL model specifically designed to predict AS events accurately. The performance of the DSC model is evaluated against existing techniques, revealing its potential to enhance the understanding and predictive power of DL algorithms in AS. It outperforms other models by achieving an average AUC score of 92%. The significance of this research lies in its contribution to identifying functional implications and potential therapeutic targets associated with AS, with applications in genomics, bioinformatics, and biomedical research. The findings of this study have the potential to advance the field and pave the way for more precise and reliable predictions of AS events, ultimately leading to a deeper understanding of genetic information processing and its impact on human physiology and disease.

## 1 Introduction

Bioinformatics, a domain that merges computer technology with molecular biology, has transformed biological research through the utilization of machine learning (ML) and deep learning (DL) models. These models have substantially enhanced various aspects, encompassing classification, feature selection, and the analysis of genetic sequences, including DNA, RNA, and proteins (Siraj et al., 2021). The foundational components of genetic information involve DNA, RNA, and protein sequences, where the flow of genetic information progresses from DNA to RNA and subsequently to proteins (Kong et al., 2021). DNA molecules are comprised of two strands with adenine (A), cytosine (C), thymine (T), and guanine (G) as the four bases (Wahab et al., 2021). Conversely, RNA is single-stranded and features uracil (U) instead of thymine (T) (Leman et al., 2020). A pivotal process in the transmission of genetic information is alternative splicing (AS), also known as differential splicing or alternative RNA splicing. AS generates multiple messenger RNA (mRNA) molecules from a single pre-mRNA molecule, resulting in the production of numerous proteins from a single gene. While reverse transcription polymerase chain reaction (RT-PCR) is a conventional method for AS prediction, it is intricate and costly. Consequently, bioinformatics researchers have turned to ML techniques for more streamlined and cost-effective predictions (Nicolaidou et al., 2020). ML algorithms have been widely applied in genomics due to their capacity to discern patterns and extract knowledge from extensive datasets (Yusuf et al., 2021). DL, a popular subfield of ML, utilizes hierarchical learning to extract increasingly abstract representations of data, enabling the learning of complex representations directly from raw data (Mahmud et al., 2021). DL models, such as Convolutional Neural Networks (CNNs), have gained acceptance in the research community due to their specificity and accuracy (Ioannis and Mpesiana, 2020). However, the performance of CNNs heavily relies on their structure, consisting of different layers, activation functions, pooling operations, initial parameter settings, and convolutional kernels (Tian et al., 2020). Splicing, a molecular biology mechanism, entails the removal of introns (non-coding regions) from pre-mRNA and the fusion of the remaining exons (coding regions) to form the final mRNA strand, a crucial step in protein synthesis. Alternative splicing (AS) brings about alterations in this process, facilitating the rearrangement of exons and resulting in a variety of mRNA molecules and a diverse array of protein products. However, genetic mutations have the potential to interfere with splicing, causing the production of deleterious proteins rather than normal ones. Certain cancers, including lung, colorectal, and breast cancer, as well as diseases like Hutchinson-Gilford progeria syndrome, which accelerates aging, are caused by these mutations. The splicing process can be made more difficult by mutations that take the form of exon skipping, alternative splice site selection, or intron retention (Louadi et al., 2019).

In recent years, DL techniques have revolutionized various domains, including genomics, image processing, and natural language processing. This paradigm shift has led to the development of innovative models and frameworks aimed at addressing complex challenges in diverse fields. In this context, the literature presents a selection of seminal works that harness DL methodologies to tackle prominent research tasks. From automated frameworks for genomic analysis to advanced models for disease identification and translation systems, these studies exemplify the versatility and efficacy of DL approaches, highlighting key advancements and contributions in various domains. Zabardast et al. (Amin et al., 2023) introduced an automated framework for conducting experiments on various models, architectures, and settings to streamline the development process and identify optimal models for RNA splice site detection. This framework facilitates the evaluation of several DL-based splice site detectors, eliminating time-consuming development efforts. Holst et al. (Holst et al., 2023) utilized DL for gene calling, aiming to develop a fully applicable, quick, and user-friendly tool for generating primary gene models from DNA sequence alone. Their approach yields state-of-the-art quality predictions, closely aligning with references on most metrics compared to other *de novo* techniques. The integration of Helixer’s predictions into pipelines or standalone usage offers further enhancements in quality. Additionally, the potential for growth and development in gene calling using DL remains substantial. Kumar et al. (Kumar et al., 2023) highlighted the utility of a reference genome in maintaining data quality while compressing FastQ files. Initially, FastQ files are divided into three streams: quality score, sequence, and identifier, with each stream utilizing a different compression method. Sharma et al. (Sharma et al., 2023) offer a comprehensive analysis of machine translation models, aiming to provide insights into various architectures, comparative evaluations, and future perspectives for translation tasks. Their study critically examines existing models in comparison to the current state-of-the-art. Agrawal et al. (Agrawal et al., 2024) introduced MultiFeNet, a CNN-based DL model, for brain tumor classification. MultiFeNet utilizes multi-scale feature scaling to extract features from magnetic resonance imaging (MRI) images. Kaur et al. (KAUR et al., 2023) introduced a deep ensemble learning model (DELM) for autonomous plant disease identification. Their study utilizes transfer learning to enhance pre-trained models and combat overfitting through various augmentation methods such as image enhancement, rotation, and scaling. The research provides a comprehensive taxonomy of single model and ensemble learning model performance in the classification of high-resolution photos of tomato plant leaf disease. Nazari et al. (Nazari et al., 2018) proposed a hybrid DL model, combining CNN and RNN, to identify the location of branch points in RNA splicing. Their model achieved impressive results on two publicly available datasets, with AUC-ROC scores of 97.29% and 96.86% and prAUC scores of 67.08% and 69.62%, respectively. An additional remarkable study conducted by Huang et al. (Huang et al., 2021) presented the WeakRM weakly supervised deep learning model. This model was designed for the detection of RNA modifications from low-resolution epitranscriptome datasets. The application of this model to three distinct types of RNA modifications demonstrated a substantial enhancement in prediction performance when compared to existing models. A CNN model specifically designed for categorizing alternative splicing patterns based on exon junction sequences was created by Louadi et al. (Louadi et al., 2019). The model displayed varying AUC scores for different inclusion levels, averaging at a substantial AUC score of 90.8%. Exon inclusion levels refer to the extent to which exons are included in a gene’s final mRNA transcript. These recent studies contribute significantly to the field of AS prediction and highlight the potential of DL models in addressing the challenges associated with RNA splicing. While previous research has made strides in accurately predicting AS events, limitations remain to overcome. For instance, existing models struggle to capture AS events in the presence of mutations that disrupt the DNA or RNA base sequence, known to contribute to multiple types of cancer. Additionally, the performance of these models, including a CNN-based approach proposed by Louadi et al. (Louadi et al., 2019), yielded relatively low AUC scores for various AS events and subsets. Hence, there exists an urgent necessity to explore the intricate layers of DL models in order to comprehend the behavior of mutations and their repercussions on AS prediction. The primary goal of this research is to augment the predictive capabilities of DL models for alternative splicing. To achieve this aim, the study will pursue the following objectives:

i. Examine cutting edge methods for alternative splicing.
ii. Build a customized deep learning model to effectively forecast AS events.
iii. Assess the performance of the proposed model in comparison to existing techniques.

AS plays a pivotal role in fundamental physiological processes like homeostasis, cell division, and tissue maintenance. Notably, AS has been linked to cancer progression and prognosis (Zhao et al., 2020), underscoring its significance in understanding and addressing complex diseases. Existing challenges in accurately predicting AS events, particularly in the presence of mutations, necessitate innovative approaches. This study is motivated by the imperative to improve the predictive capabilities of DL models in AS, aiming to unravel functional implications and potential therapeutic targets associated with AS, thereby advancing our understanding of genetic information processing and its implications for human health. By achieving the above objectives, the contributions of this study are:

• Introduction of the deep splicing code (DSC) model, a novel deep learning-based approach for AS prediction.
• Evaluation of DSC against existing techniques, demonstrating superior performance with an average AUC score of 92%.
• Advancement in the field by enhancing the precision and reliability of AS event predictions.
• Potential applications in genomics, bioinformatics, and biomedical research, offering a valuable tool for researchers and clinicians.
• Identification of functional implications and potential therapeutic targets associated with AS, aiding in the broader understanding of genetic information processing and its impact on human physiology and disease.

Section 2 discusses the method and methodology used. Section 3 presents the results.

## 2 Materials and methods

**FIGURE 1:**
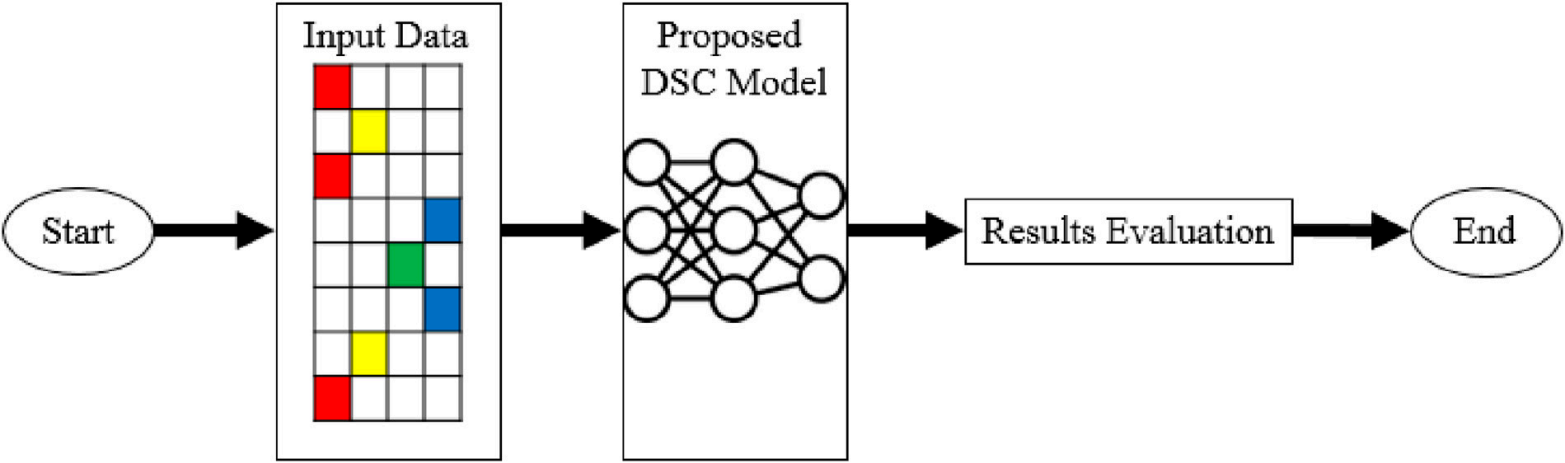
Proposed methodology.

The intricate methodology employed in the current study is described in this section. The goal is to present a comprehensive explanation of the recommended methodology, beginning with an overview of the testing dataset. The configuration of the computational environment will be described in detail, with an emphasis on the use of the Google Colab platform and the benefits of using a Tesla T4 GPU to boost computational capacity. After that, we will discuss the deep learning-based model DSC in detail as it is the primary focus of this work. This section will also address the evaluation methodology, highlighting the significance of the Area under the ROC (AUC-ROC) curve as the primary evaluation statistic. By making these important elements clear, we intend to lay a strong foundation for a careful and comprehensive analysis of our research objectives and conclusions. The suggested methodology is illustrated visually in Figure 1.

### 2.1 Dataset selection

The utilized dataset consists of human internal exons and has undergone preprocessing as detailed by Louadi et al. (2019). It comprises three categories of alternative splicing (AS) events: Exon Skipping (ES), Alternative 3’ (Alt3), and Alternative 5’ (Alt5) splice sites. The dataset features three variables representing the length of exons and neighboring introns. This dataset was chosen due to its comprehensive coverage of diverse AS events in the human genome, ensuring the model’s ability to generalize across various biological contexts. Table 1 provides a summary of the dataset, offering insights into its characteristics and composition.

**TABLE 1:** Dataset statistics.

As types	HEvents	MREvents
Alternative 3’	1,388	944
Alternative 5’	1,568	602
Exon Skipping	4,952	6,838

### 2.2 Preprocessing steps

Preprocessing of sequence data for modeling involved employing a one-hot-encoding technique, resulting in a 140 × 4 matrix representation. This technique converts categorical variables, such as nucleotide sequences, into a numerical format suitable for input into machine learning models. The implementation of the deep learning-based CNN model was carried out in Python using Google Colab. During the training process, a 10-fold cross-validation strategy was employed to assess the model’s performance. Ten folds with identical sizes were randomly selected from the dataset. The models were trained on eight of these folds, allowing them to identify patterns and connections in the data. An additional fold was designated for early stopping to prevent overfitting and enhance model performance. Regular monitoring of the early stopping fold occurred, halting the training process if the model’s performance did not surpass a predefined threshold. Finally, the remaining fold was used as the test set to independently assess the model’s performance on unseen data. This method ensured a thorough evaluation of the model’s generalization ability and accuracy in making predictions across various subsets of the dataset. Throughout the training process, a 10-fold cross-validation strategy was employed. Ten folds with identical sizes were randomly selected from the dataset. The models were trained on eight of these folds, which helped them identify patterns and connections in the data. An additional fold was designated for early stopping to prevent overfitting and enhance model performance. Regular monitoring of the early stopping fold took place, and if the model’s performance did not surpass a predefined threshold, the training process was halted. Finally, the remaining fold was used as the test set so that the model’s performance could be independently assessed on data that had not yet been seen. This method of partitioning the dataset ensured a thorough assessment of performance by taking into account several dataset subsets. It comprehensively gauged the model’s generalization ability and accuracy in making predictions.

Cross-validation is a powerful technique used in machine learning and statistical analysis to assess models’ performance and generalization ability. In this research, we employed the widely used 10-fold cross-validation method to ensure reliable and robust evaluations of our DL-based model for predicting AS events. Within the context of 10-fold cross-validation, the dataset undergoes division into ten equally sized subsets or folds. Throughout the training stage, the model undergoes training on eight folds, facilitating the acquisition of intricate patterns and relationships within the data. The retention of the remaining fold is designated for early stopping, involving consistent monitoring of the model’s progress. Should the performance of the early stopping fold not exhibit improvement beyond a predefined threshold, the training process is ceased to forestall overfitting. Once the training phase is complete, the model is tested on the fold kept aside during training, serving as an independent evaluation set. This test set provides an unbiased assessment of the model’s performance on never-before-seen data, demonstrating its ability to generalize and produce precise predictions. By employing 10-fold cross-validation, we ensure comprehensive evaluations of our DL model’s performance, considering different subsets of the dataset. This approach allows us to obtain reliable performance metrics and gain insights into the model’s effectiveness in predicting AS events. It enhances confidence in the model’s performance by validating its performance across multiple subsets of the data, ultimately contributing to our research findings’ overall reliability and robustness.

### 2.3 DSC architecture

**FIGURE 2:**
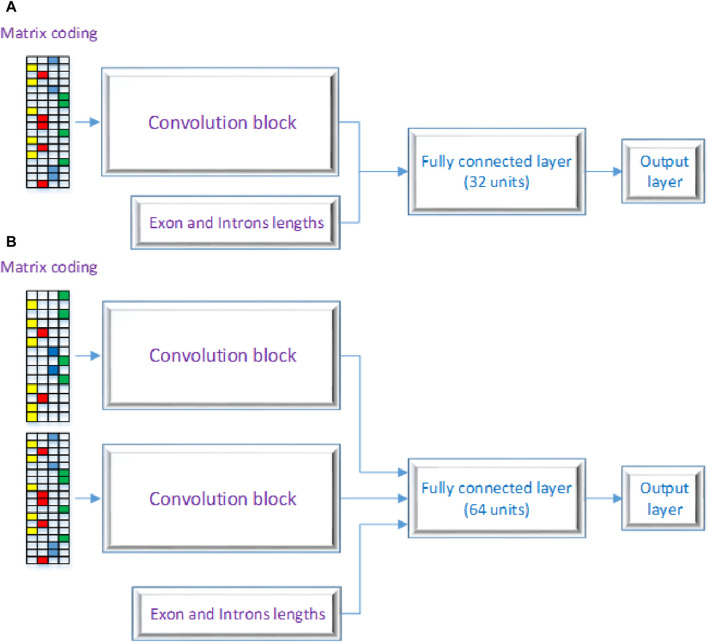
Proposed DSC Architecture with **(A)** a a single layer and **(B)** as a multiple layer architecture.

Figure 2 displays the CNN’s structure that was used in this investigation, comprising three primary layers: the convolutional block, the fully connected layers, and the output layer. CNNs are inspired by the architecture of the visual system and its computational models, emphasizing local connectivity between neurons and hierarchically organized image transformations. This design allows CNNs to achieve translational invariance by employing neurons with identical parameters for patches from the preceding layer at different locations. The foundational work by Yann LeCun and his team (LeCun et al., 1998) utilized the error gradient to develop CNNs, showcasing remarkable performance in various pattern recognition applications. Our proposed model encompasses two architectures, labeled as 2 (A) and 2 (B) as shown in Figure 2. One RNA sequence is the input for the first architecture, while two RNA sequences are the input for the second architecture. As seen in Figures 2A, B, these designs contain three different kinds of neural layers: output layers, fully connected layers, and convolutional layers. Each layer in the CNN has a distinct purpose.

In the convolutional block, many kernels convolve over the whole picture and intermediate feature maps, producing a variety of feature maps. This method replaces fully connected layers and has the advantage of faster learning times. Neural networks that employ fully connected layers for higher-order reasoning come after the convolutional and pooling layers. Every neuron in these layers is linked to every activation in the layer below, and the activation of each neuron is calculated by multiplying a matrix with a bias offset. The 2D feature maps from the preceding layers are transformed into a 1D feature vector using completely connected layers. After that, this vector can be used for categorization or subjected to additional processing. Basic ideas like spatial subsampling, linked weights, and local receptive fields are all incorporated into the CNN design. Convolutional layer neurons extract basic visual components like edges and corners by receiving inputs from adjacent units in the layer below. These retrieved features are combined with subsequent convolutional layers to discover higher-order features. With tied weights, several units share the same weights since it is anticipated that elementary feature detectors that are successful for a section of an image will be useful throughout the full image. A convolutional layer’s units are arranged in planes and use the same set of weights to build distinct features for each unit. Multiple feature maps can be created at each place since the feature maps collectively represent the results of these planes. The output layer, the final layer of the CNN, receives input from the preceding layers, performs calculations through its neurons, and produces an output. It plays a crucial role in generating the desired predictions or classifications. The proposed model architecture employs a dual-branch structure, each branch processing a distinct input. To introduce non-linearity, both branches start with a rectified linear unit (ReLU) activation function, a regularization dropout layer, and a convolutional layer. The features are then downsampled by applying max-pooling layers with a pool size of 2 and a stride of 2 after each convolutional layer. The first branch comprises four convolutional layers, sequentially increasing the number of filters (8, 16, 32, and 64) and utilizing corresponding kernel sizes (7, 4, 3, and 2). The second branch mirrors the architecture of the first branch. The outputs of both branches are then concatenated to capture the combined information. The multi-dimensional data is flattened and then combined with a third input to create a one-dimensional feature vector. The resulting combined features are then input into a dense layer that has 1,024 units and a rectified linear unit (ReLU) activation function to help the model capture complex patterns and representations. After the dense layer, a dropout layer with a dropout rate of 0.5 is used to prevent overfitting. The model’s final layer is made up of a dense layer with a sigmoid activation function and a single unit. With this design, binary categorization is made easier by producing a probability score that falls between 0 and 1. The binary cross-entropy loss function, which is especially useful for applications involving binary classification, is used by the model during training. With a learning rate of 0.0005, beta parameters of 0.9 and 0.999, an epsilon value of 1e-08, and no decay, the Adam optimizer is used. Accuracy is used to evaluate the model’s performance by measuring the percentage of correctly categorized samples and assessing the model’s classification power. The details of each layer are broken down as follows:

#### 2.3.1 Convolutional layer

The convolutional layer applies a set of filters to the input data and performs a convolution operation. For each filter, the output feature map is calcualted using Eq. 1.

$$output_{i}=\underset{j}{\sum }\left(input_{ij}.filter_{j}\right)+bias$$ (1)

#### 2.3.2 Dropout layer

The dropout layer helps prevent overfitting by randomly dropping out a fraction of the input units during training. Eq. 2 represents the dropout layer.

$$output_{i}=\left\{\begin{array}{cc} input_{i}.mask, & ifmask_{i}=1\\ 0, & otherwise \end{array}\right.$$ (2)

#### 2.3.3 Activation function

The activation function introduces non-linearity into the model, enabling it to learn complex relationships. In this model, the ReLU activation function is calculated as Eq. 3.

$$output=max\left(0,input\right)$$ (3)

#### 2.3.4 Max pooling layer

The max pooling layer downsamples the spatial dimensions of the input feature maps by selecting the maximum value within a pooling window. The Eq. 4 calculates the max pooling layer.

$$output_{i}=max\left(input_{i}^{p}\right)$$ (4)

#### 2.3.5 Fully connected layer

The neural network employs fully connected layers for higher-order reasoning, following a series of convolutional and pooling layers. A fully connected layer’s neurons are connected to the activations of the layers below them. Neuron activations are computed by multiplying matrices with a bias offset. The 2D feature maps from previous levels are transformed into a 1D feature vector using these fully connected layers. This final vector can be utilized as a feature vector for additional processing or input into a collection of categories for classification. Three fundamental concepts underpin CNN architecture: shared weights, local receptive fields, and spatial subsampling. Based on their local receptive fields, nearby units in the layer below provide inputs to each unit in a convolutional layer. This makes it possible for neurons to distinguish basic visual components like edges and corners. These retrieved features are combined with subsequent convolutional layers to discover higher-level, more complicated features. Tied weights, another name for shared weights, assume that basic feature detectors that work well in one area of an image will work similarly well in the entire image. As a result, a collection of units—referred to as tied weights—shares the same set of weights. A convolutional layer’s units are organized into planes, and each plane builds a certain feature using the same set of weights. The products of these planes, or feature maps, serve as the fundamental building blocks of every convolutional layer in the network, enabling the production of multiple feature maps at each node (Voulodimos et al., 2018). The fully connected layer is calculated as Eq. 5.

$$output=activation\left(input.weights+bias\right)$$ (5)

#### 2.3.6 Output layer

The output layer employs a sigmoid activation function to generate a probability value within the range of 0 and 1, indicating the likelihood of the input belonging to a particular class. The formula for output layer is shown in Eq. 6.

$$output=\sigma \left(input.weights+bias\right)$$ (6)

#### 2.3.7 Model loss

The binary cross-entropy loss function is employed by the model. When classifying inputs into one of two classes is the goal of a binary classification task, binary cross-entropy is a frequently used metric. The difference between the actual labels and the expected probabilities is measured by this metric. To calculate the binary cross-entropy loss, utilizes Eq. 7.

$$Loss=-\frac{1}{N}\overset{N}{\underset{i=1}{\sum }}\left[y_{i}log\left({\hat{y}}_{i}\right)+\left(1-y_{i}\right)log\left(1-\hat{y}\right)\right]$$ (7)

Where:

- *N* is the number of samples in the dataset.
- *y*_*i*_ is the true label of the *i*-th sample (either 0 or 1).
- ${\hat{y}}_{i}$ is the predicted probability of the *i*-th sample belonging to class 1.

Adam, a popular optimization algorithm for training deep neural networks, is used to minimize this loss function. It adapts the learning rate based on the gradients to speed up convergence. By minimizing the binary cross-entropy loss using the Adam optimizer, this model aims to learn the optimal set of weights and biases that can accurately classify the inputs into their respective classes.

#### 2.3.8 Model summary

The proposed model is a DL architecture designed for AS prediction. It takes three types of inputs: “inputs1” and “inputs2” represent sequences with four features per element, and “inputs3” represents an additional input with three features. The model consists of eight hidden layers, with four layers for each input. Each hidden layer consists of a convolutional layer (“Conv1D”) followed by a dropout layer for regularization and an activation layer using the ReLU activation function. Max pooling layers (“MaxPooling1D”) are incorporated to downsample the spatial dimensions of the feature maps. The convolutional layers have different filter sizes and kernel sizes, enabling the model to capture varying levels of information and patterns. The outputs from the convolutional layers are then concatenated using the “keras.layers.concatenate” function. This concatenated representation is flattened and combined with “inputs3” through another concatenation operation. This combined representation is passed through a fully connected layer (“Dense”) with 1,024 units and ReLU activation. Dropout is applied to this layer to prevent overfitting and enhance generalization. The final output layer consists of a single unit with a sigmoid activation function, making the model suitable for binary classification tasks. The model is trained using the binary cross-entropy loss function and the Adam optimizer with a learning rate 0.0005. The proposed model demonstrates improved performance compared to existing models for AS prediction. It balances model capacity and computational efficiency using eight hidden layers, achieving high AUC scores. The model’s architecture and parameters have been fine-tuned through extensive experimentation to optimize its predictive performance on the given task. The following Table 2 presents the model summary.

**TABLE 2:** Model summary.

Layer (type)	Output shape	Param #	Connected to
input_5 (InputLayer)	[(None, 140, 4)]	0	[]
input_4 (InputLayer)	[(None, 140, 4)]	0	[]
conv1d_12 (Conv1D)	(None, 134, 8)	232	[“input_5[0][0]”]
conv1d_8 (Conv1D)	(None, 134, 8)	232	[“input_4[0][0]”]
dropout_13 (Dropout)	(None, 134, 8)	0	[“conv1d_12[0][0]”]
dropout_9 (Dropout)	(None, 134, 8)	0	[“conv1d_8[0][0]”]
activation_12 (Activation)	(None, 134, 8)	0	[“dropout_13[0][0]”]
activation_8 (Activation)	(None, 134, 8)	0	[“dropout_9[0][0]”]
max_pooling1d_12 (MaxPooling1D)	(None, 67, 8)	0	[“activation_12[0][0]”]
max_pooling1d_8 (MaxPooling1D)	(None, 67, 8)	0	[“activation_8[0][0]”]
conv1d_13 (Conv1D)	(None, 64, 16)	528	[“max_pooling1d_12[0][0]”]
conv1d_9 (Conv1D)	(None, 64, 16)	528	[“max_pooling1d_8[0][0]”]
dropout_14 (Dropout)	(None, 64, 16)	0	[“conv1d_13[0][0]”]
dropout_10 (Dropout)	(None, 64, 16)	0	[“conv1d_9[0][0]”]
activation_13 (Activation)	(None, 64, 16)	0	[’dropout_14[0][0]”]
activation_9 (Activation)	(None, 64, 16)	0	[“dropout_10[0][0]”]
max_pooling1d_13 (MaxPooling1D)	(None, 32, 16)	0	[“activation_13[0][0]”]
max_pooling1d_9 (MaxPooling1D)	(None, 32, 16)	0	[“activation_9[0][0]”]
conv1d_14 (Conv1D)	(None, 30, 32)	1,568	[’max_pooling1d_13[0][0]”]
conv1d_10 (Conv1D)	(None, 30, 32)	1,568	[“max_pooling1d_9[0][0]”]
dropout_15 (Dropout)	(None, 30, 32)	0	[“conv1d_14[0][0]”]
dropout_11 (Dropout)	(None, 30, 32)	0	[“conv1d_10[0][0]”]
activation_14 (Activation)	(None, 30, 32)	0	[“dropout_15[0][0]”]
activation_10 (Activation)	(None, 30, 32)	0	[“dropout_11[0][0]”]
max_pooling1d_14 (MaxPooling1D)	(None, 15, 32)	0	[“activation_14[0][0]”]
max_pooling1d_10 (MaxPooling1D)	(None, 15, 32)	0	[“activation_10[0][0]”]
conv1d_15 (Conv1D)	(None, 14, 64)	4,160	[“max_pooling1d_14[0][0]”]
conv1d_11 (Conv1D)	(None, 14, 64)	4,160	[“max_pooling1d_10[0][0]”]
dropout_16 (Dropout)	(None, 14, 64)	0	[“conv1d_15[0][0]”]
dropout_12 (Dropout)	(None, 14, 64)	0	[“conv1d_11[0][0]”]
activation_15 (Activation)	(None, 14, 64)	0	[“dropout_16[0][0]”]
activation_11 (Activation)	(None, 14, 64)	0	[“dropout_12[0][0]”]
max_pooling1d_15 (MaxPooling1D)	(None, 7, 64)	0	[“activation_15[0][0]’]
max_pooling1d_11 (MaxPooling1D)	(None, 7, 64)	0	[“activation_11[0][0]”]
concatenate_2 (Concatenate)	(None, 7, 128)	0	[“max_pooling1d_15[0][0]”, “max_pooling1d_11[0][0]”]
flatten_1 (Flatten)	(None, 896)	0	[“concatenate_2[0][0]”]
input_6 (InputLayer)	[(None, 3)]	0	[]
concatenate_3 (Concatenate)	(None, 899)	0	[“flatten_1[0][0],” “input_6[0][0]”]
dense_2 (Dense)	(None, 1,024)	921,600	[“concatenate_3[0][0]”]
dropout_17 (Dropout)	(None, 1,024)	0	[“dense_2[0][0]”]
dense_3 (Dense)	(None, 1)	1,025	[“dropout_17[0][0]”]

### 2.4 Results evaluation

Our research model’s performance evaluation relies on a pivotal metric known as the Area Under the Curve (AUC). AUC is a widely acknowledged and extensively utilized evaluation measure in deep learning and predictive modeling. It imparts valuable insights into the model’s proficiency in distinguishing between positive and negative instances, rendering it particularly suitable for classification tasks. AUC scrutinizes the quality of the model’s predictions across various decision thresholds, capturing the trade-off between the true positive rate and false positive rate. The AUC score spans from 0 to 1, with a higher value signifying superior discrimination power and classification accuracy. By employing AUC as our primary evaluation metric, we can effectively measure the predictive capability of our deep learning model for alternative splicing events. A higher AUC score indicates a more accurate and reliable model, showcasing its potential contribution to advancements in bioinformatics, genomics, and biomedical research.

## 3 Experiments and results

This section offers comprehensive details, which entails enhancing the CNN-based DSC model’s predictive capabilities for AS event forecasts. Compared to the original model’s six layers, the research presents a modified model with an original architectural layout made up of eight layers. After giving considerable thought to the model’s capability, computational complexity, and overfitting prevention, the choice was reached to include eight layers. After experimenting with several layer configurations, we found that there were diminishing returns after 8 layers, suggesting that 8 layers was the ideal combination of model depth and performance. We evaluated the two models’ performances throughout a range of epochs, from 10 to 200, in order to fully compare them. It should be noted that the average AUC-ROC scores served as our main performance metric. Through this comparative investigation, we were able to determine that the updated 8-layer model outperformed the original 6-layer design in terms of prediction. We highlight the efficacy of our suggested improvement and its capacity to outperform the current model by presenting and analyzing these comparison data. This section provides a critical connection between the original problem description and the thorough analysis of our suggested solution, demonstrating how the eight-layer modified CNN model skillfully tackles the difficulties given by mutations in AS event predictions.

The DSC model, a deep learning framework based on CNN built in Python 3.10, a language commonly used in the machine learning area, was the means by which the research was carried out. We leveraged the power of the Google Colab platform for this inquiry, which offered an intuitive virtual environment with powerful hardware resources. Colab’s computing efficiency was significantly increased by the easy incorporation of the Tesla T4 GPU’s processing power into the environment. Additionally, the abundance of pre-installed packages and open accessibility of Google Colab provided researchers with a simple way to browse and execute the recommended methodology with exceptional efficacy.

This section presents the results of our experiments conducted with the proposed DSC model, followed by a comparison with the existing model (Louadi et al., 2019). The results highlight the enhanced performance of the proposed model, which achieves higher average AUC scores across various inclusion levels. Throughout our research, we explored various modifications to the existing model, including adjustments to kernel sizes, strides and incorporating a hybrid CNN model with DT and SVM. We also experimented with activation functions such as ReLU, Sigmoid, and Tanh. However, these modifications yielded fewer results compared to the existing model. We experimented with different layer depths using the human internal exon dataset for AS prediction to determine an optimal configuration. We observed that increasing the layer depth beyond eight resulted in diminishing returns in performance improvement. Deeper models offered increased capacity but also introduced additional computational complexity and a higher risk of overfitting. Conversely, shallower models with fewer than eight layers demonstrated a decline in performance, as they needed to capture the intricate patterns and relationships in the dataset, leading to underfitting. Given the intrinsic complexity of the human internal exon dataset, we opted for the model with eight hidden layers (four layers for each input) to achieve a balance between capacity and computational efficiency. The model has 32 filters with a first layer kernel size of 7, 8 filters with a second layer kernel size of 4, 8 filters with a third layer kernel size of 3, and 8 filters with a final layer kernel size of 1. The strides remain consistent at 1 across all layers for both inputs. We trained the model for various epochs using the same approach as the existing model. The proposed model demonstrated improved performance by comparing the results with the existing model.

**FIGURE 3:**
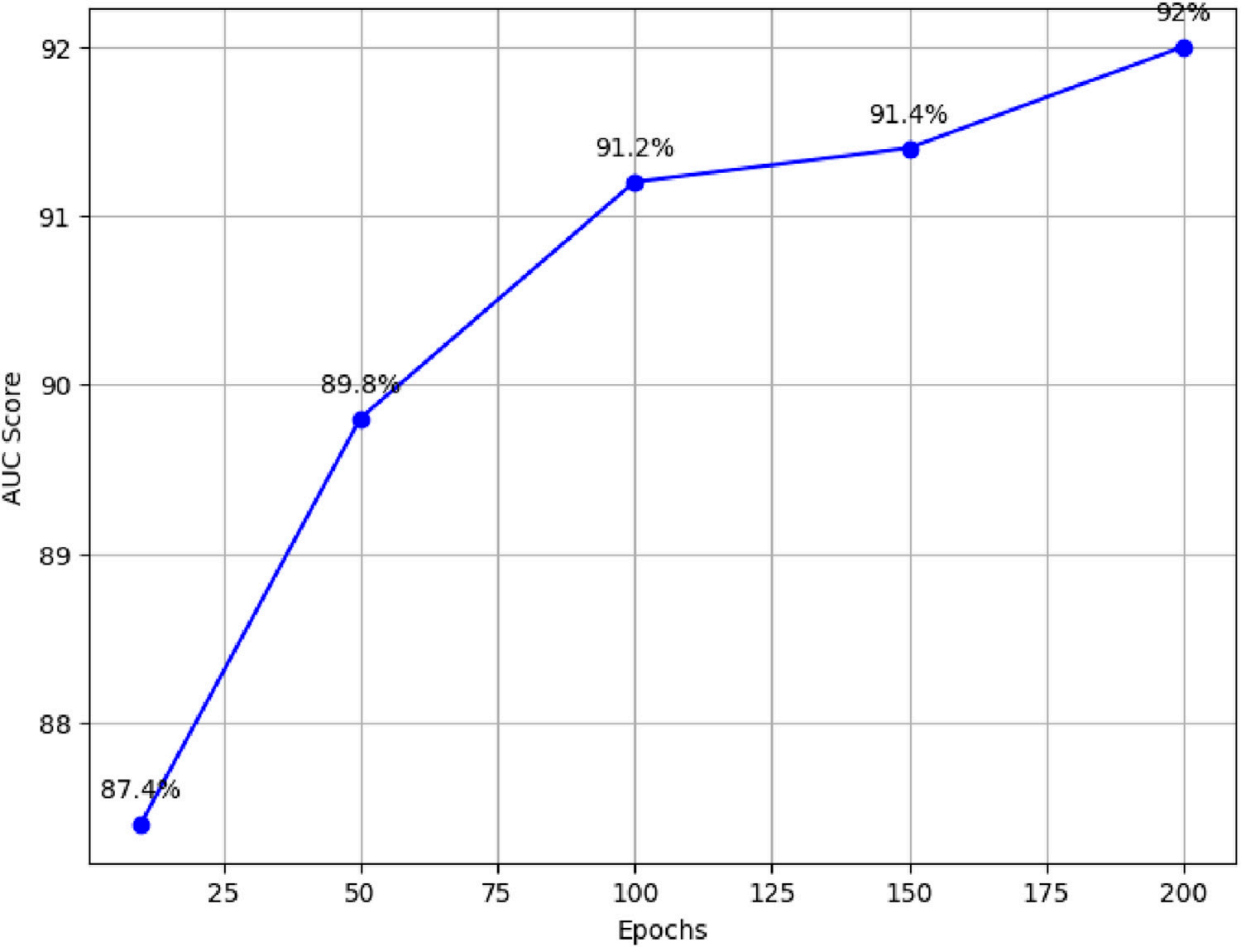
Performance comparison of proposed model across different epochs.

For example, with 10 epochs, the proposed model achieved an average AUC score of 87.4%. Furthermore, training the model for 50 epochs resulted in an average AUC score of 89.8%. For 100 epochs, the model achieved an average AUC score of 91.2%. Similarly, training the model for 150 epochs resulted in an average AUC score of 91.4%. Finally, the model was trained for 200 epochs, resulting in an average AUC score of 92%, as illustrated in Figure 3.

### 3.1 Comparison with existing DSC model

The model described in (Louadi et al., 2019) consists of six hidden layers. Using the current model, we ran numerous tests at epoch intervals of 10, 50, 100, 150, and 200. The model setup employed by the authors made use of particular parameters. Three convolutional layers were added to each block as part of their methodology. The first layer consisted of 32 filters with a window size of seven units; the second layer included 8 filters with a window size of four units; and the last layer had 8 filters with a window size of three units. For both inputs, a stride of 1 was used in all layers. They used a Rectified Linear Unit (ReLU) to apply an activation layer after each convolutional layer. A dropout layer with a probability of 0.2 was added to address overfitting. They also included a max-pooling layer with a 2 unit stride and window size. The architecture and performance of the model were greatly impacted by these parameters.

**FIGURE 4:**
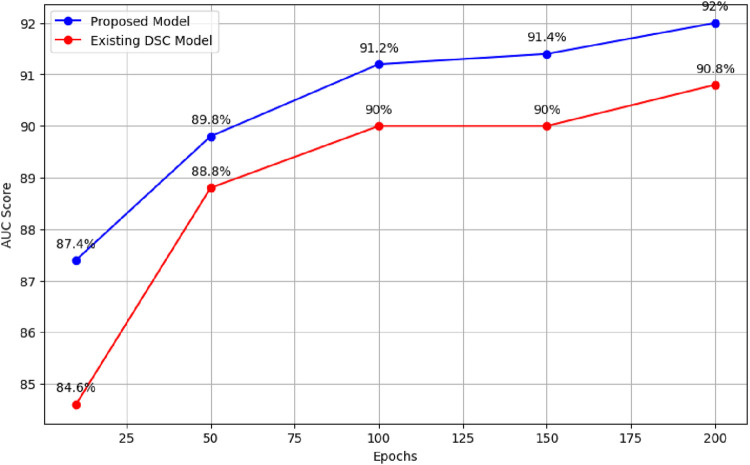
Performance comparison of proposed model and existing model across different epochs.

When the existing model was trained for 10 epochs, varying AUC scores were obtained, resulting in an average score of 84.6%. When trained over distinct epochs, the current model yields inconsistent results. Interestingly, it performed better than the model trained for 10 epochs after 50 epochs of training on the provided dataset. Different results were obtained, it achieved an average AUC score of 88.8%. The researchers noticed better performance when training the current model with 100 epochs as opposed to the model trained with 50 epochs. In particular, they obtained an average AUC score of 90%. In a similar vein, after training for 150 epochs, the current model obtained an average AUC score of 90%. During our experiments with the existing model, the most noteworthy outcome was observed when the model was trained for 200 epochs, surpassing the results of all previous training runs with 10, 50, 100, and 150 epochs. The average AUC score attained by the existing model after 200 epochs was 90.8%. Figure 4 presents comparison of the proposed model with the existing model across different epochs.

These results demonstrate the superiority of the proposed model, which outperforms the existing model (Louadi et al., 2019) in terms of AUC score. The findings support the effectiveness of the proposed model architecture and parameter configurations in handling the complexities of AS prediction. Table 3 summarises the results for both the existing and updated models.

**TABLE 3:** Updated vs. existing model’s result summary.

Epochs	Average AUC	
	Existing model	Updated model
10	84.6	87.4
50	88.8	89.8
100	90	91.2
150	90	91.4
200	90.8	92

Our study introduces the DSC model, leveraging DL techniques to enhance the prediction of AS events. By addressing the limitations of existing approaches, the DSC model achieves superior predictive performance, with an average AUC score of 92%. This innovative model aims to uncover disease-associated splice variants and elucidate the regulatory mechanisms underlying AS, offering valuable insights into the molecular basis of various diseases, including cancer. Through collaborative efforts with clinicians, biologists, and pharmaceutical companies, the DSC model’s predictions can be validated in clinical or experimental settings, translating into tangible outcomes for patient care and biomedical research. Thus, our research not only advances the field of AS prediction but also holds promise for improving disease diagnosis and treatment strategies, ultimately contributing to a deeper understanding of genetic information processing and its impact on human health. The Table 4 summarizes the performance of DeepSplice compared to these models across the selected metrics:

**TABLE 4:** Comparison with the existing state-of-the-art models from the literature.

Model	AUC score (%)	Precision	Recall	F1 score
DeepSplice	92	0.90	0.89	0.895
Siraj et al. (2021)	85	0.83	0.81	0.820
Kong et al. (2021)	88	0.86	0.84	0.850
Wahab et al. (2021)	90	0.87	0.88	0.875
Leman et al. (2020)	89	0.88	0.87	0.875

The findings of this study hold significant implications for real-world genomic research and the understanding of AS. By introducing the DSC model, our research offers a promising avenue for advancing the field of AS prediction. Beyond reporting improved performance metrics, the practical implications of these advancements are manifold. Firstly, the DSC model’s enhanced predictive ability holds promise for identifying disease-associated splice variants and elucidating the regulatory mechanisms underlying AS events, thus offering valuable insights into the molecular basis of various diseases, including cancer. Furthermore, the model’s potential clinical relevance is underscored by its capacity to aid in disease diagnosis and prognosis, guide personalized medicine approaches, and identify biomarkers for disease monitoring. Collaborative efforts with clinicians, biologists, and pharmaceutical companies can further validate the model’s predictions in clinical or experimental settings, translating them into tangible outcomes for patient care and biomedical research.

## 4 Conclusion

Alternative splicing (AS) is a crucial process in which exons, the coding regions of genes, are rearranged to produce various mature messenger RNAs, producing multiple proteins from a single gene. Disruptions in AS can lead to various diseases, including brain tumours, lung cancer, and breast cancer. In this study, we examined different ML and DL techniques employed for accurate and efficient AS prediction. While these techniques have shown promising results, the reported average AUC score of 90.8% indicates room for improvement. One of the major challenges lies in the detection of the remaining 9.2% of AS events that were missed by the applied techniques, potentially leading to the misdiagnosis of cancerous cases. To address this, we proposed a DL-based model CNN. Our study concentrated on the CNN model’s deep layers to improve its capacity for AS prediction. The suggested model was tested on a publicly accessible dataset of human internal exons after being built in Python using the Google Colab platform. Through numerous experiments, we observed that adding two hidden layers resulted in eight hidden layers significantly improved the model’s performance compared to the existing approaches. Our proposed model achieved an excellent result, surpassing the existing model and increasing the average AUC score from 90.8% to 92%. This improvement reduces the risk of missing cancerous cases, making our proposed model highly valuable for cancer prognosis research and related stakeholders.

## 5 Future work

To address the need for further exploration into scalability and adaptability aspects to solidify DeepSplice’s foundational and forward-looking contributions, future research should focus on several key areas. Firstly, investigating the integration of multi-omics data, including gene expression profiles and epigenetic changes, can enhance predictive models for AS and yield insightful information. Incorporating additional molecular information has the potential to uncover hidden patterns and improve the accuracy of AS prediction models. Secondly, developing ensemble models that combine different algorithms or architectures holds great potential for further improving prediction accuracy. By leveraging the strengths of multiple models, the risk of false positives or negatives in AS identification can be reduced. Lastly, using transfer learning strategies can help with AS prediction in novel biological settings. Leveraging knowledge from pre-trained models on large-scale datasets can enhance the efficiency and effectiveness of AS prediction on limited or specific datasets. Addressing these areas of future work will contribute to advancing the field, further our understanding of the role of AS in diseases, and ultimately improve diagnostic and prognostic capabilities in cancer research and beyond.

## Data Availability

The data and code presented in the study are deposited in the GitHub repository, at https://github.com/Didar-Hussain/AS-Prediction-Using-DL.
